# Beneficial effects of SS-31 peptide on cardiac mitochondrial dysfunction in tafazzin knockdown mice

**DOI:** 10.1038/s41598-022-24231-4

**Published:** 2022-11-18

**Authors:** Silvia Russo, Domenico De Rasmo, Anna Signorile, Angela Corcelli, Simona Lobasso

**Affiliations:** 1 Department of Translational Biomedicine and Neuroscience, University of Bari Aldo Moro, Bari, Italy; 2 CNR-Institute of Biomembranes, Bioenergetics and Molecular Biotechnologies, Bari, Italy

**Keywords:** Experimental models of disease, Membrane lipids

## Abstract

Barth Syndrome (BTHS), a genetic disease associated with early-onset cardioskeletal myopathy, is caused by loss-of-function mutations of the *TAFAZZIN* gene, which is responsible for remodeling the mitochondrial phospholipid cardiolipin (CL). Deregulation of CL biosynthesis and maturation in BTHS mitochondria result in a dramatically increased monolysocardiolipin (MLCL)/CL ratio associated with bioenergetic dysfunction. One of the most promising therapeutic approaches for BTHS includes the mitochondria-targeted tetrapeptide SS-31, which interacts with CL. Here, we used *TAFAZZIN* knockdown (Taz^KD^) mice to investigate for the first time whether in vivo administration of SS-31 could affect phospholipid profiles and mitochondrial dysfunction. The CL fingerprinting of Taz^KD^ cardiac mitochondria obtained by MALDI-TOF/MS revealed the typical lipid changes associated with BTHS. Taz^KD^ mitochondria showed lower respiratory rates in state 3 and 4 together with a decreased in maximal respiratory rates. Treatment of Taz^KD^ mice with SS-31 improved mitochondrial respiratory capacity and promoted supercomplex organization, without affecting the MLCL/CL ratio. We hypothesize that SS-31 exerts its effect by influencing the function of the respiratory chain rather than affecting CL directly. In conclusion, our results indicate that SS-31 have beneficial effects on improving cardiac mitochondrial dysfunction in a BTHS animal model, suggesting the peptide as future pharmacologic agent for therapy.

## Introduction

Barth syndrome (MIM302060, abbreviated BTHS) is a rare, life-threatening, X-linked recessive disease, characterized mainly by infancy or childhood-onset cardiomyopathy, skeletal myopathy, neutropenia, growth retardation, and often 3-methylglutaconic aciduria (3-MGCA)^1–4^.

The disease results from loss-of-function mutations of the *TAFAZZIN* gene, located on chromosome Xq28.12^5,6^. The major consequence is the deficient remodeling and maturation of the phospholipid cardiolipin (CL), a metabolic process that normally leads to the mature acyl composition of the molecule^7,8^. The *TAFAZZIN* gene encodes the protein tafazzin, a transacylase enzyme that transfers acyl groups from phospholipids such as phosphatidylethanolamine (PE) and phosphatidylcholine (PC) to a specific lysophospholipid (monolysocardiolipin, MLCL) during CL remodeling^9^.

CL, the signature phospholipid of mitochondrial membranes, is a key player important for several activities such as oxidative phosphorylation (i.e., energy metabolism), respiratory supercomplexes formation, protein import, and the fission–fusion cycle^10–15^.

*TAFAZZIN* mutations in BTHS result in important alterations in the level and molecular composition of CL (and MLCL)^16^. Thus, CL remodeling impairments lead to specific abnormalities in mitochondrial phospholipid composition: a reduced content of mature CL (CLm), increased levels of MLCL and altered CL acyl composition (i.e. immature CL, CLi)^17^. The tissue-specific acyl chain composition of CL is important for optimal mitochondrial function^10^. In particular, tetralinoleoyl-CL (containing four linoleic acids as acyl groups) is the most abundant mature CL form in heart and skeletal muscle, while more unsaturated fatty acids, such as arachidonic and docosahexaenoic acids, are mainly found in brain CL^18^.

Deletion of the *TAFAZZIN* gene makes CL unstable and prone to degradation^19^ and causes a decrease in the density of inner membrane proteins, suggesting that the membrane cannot accommodate large amounts of proteins unless CL is remodeled^20^. Defective tafazzin protein and CL alterations are associated with abnormal mitochondrial membrane ultrastructure and respiratory chain dysfunction^8,17^.

Like other genetic rare diseases, BTHS is underdiagnosed, with approximately 250 known cases worldwide and a prevalence of one case per million males^2,21^. Historically, the diagnosis of BTHS has been made by identification of the main clinical symptoms (mainly dilated cardiomyopathy and skeletal muscles weakness), in association with recurrent neutropenia and 3-MGCA, and then confirmed by detection of *TAFAZZIN* mutations. It is widely accepted that measurement of the elevated MLCL/CL ratio in blood spots and other tissues from patients is 100% specific and sensitive for diagnosing BTHS^22–24^. A novel MALDI-TOF/MS method for lipid analysis of intact white blood cells has been developed for a simple test of the relative amounts of CL and MLCL species. This assay consists of a single run of MS analysis, that measures the decrease in CLm levels but also takes into account the increase in CLi content, which together with the presence of MLCL is the specific fingerprint of tafazzin deficiency^25,26^. The aberrant CL metabolism leads to the disruption of mitochondrial bioenergetics and the development of the pathology and it is noteworthy that BTHS is the only human known genetic disease that is related directly to CL remodeling impairment^3^. Although BTHS is a multisystem disorder, heart failure and cardiomyopathy are the most common causes of death in BTHS^2,4^. It is well known that cardiac metabolism is highly dependent on the oxidative phosphorylation (OXPHOS) system as the primary source of energy production. Therefore, alteration of CL composition leads to impaired energy metabolism of BTHS mitochondria associated with increased production of reactive oxygen species (ROS), respiratory chain instability, and alterations in mitochondrial dynamics and biogenesis^27–29^. Previous studies have shown that both cardiomyocytes and cardiac mitochondria isolated from tafazzin-deficient mice (defined here as Taz^KD^ mice) present a reduction in fatty acid oxidation^30,31^. In addition, recent findings have demonstrated that myocardial glucose oxidation is markedly impaired in isolated working hearts from Taz^KD^ mice, which is associated with decreased pyruvate dehydrogenase activity^32^.

Although tafazzin-deficient hearts exhibit CL alterations similar to BTHS patients (i.e. high MLCL/CL ratio), it is important to underlie that Taz^KD^ mice present relatively mild and late-onset cardiomyopathy phenotypes^31,33–35^.

The development of an effective and specific therapy for BTHS patients remains challenging, particularly because of the limited number of diagnosed patients, extraordinary phenotype variability, and unpredictable clinical course. One of the most promising therapeutic approaches includes the CL-targeted tetrapeptide SS-31, which is hypothesized to improve mitochondrial inner membrane stability and bioenergetic functions^36,37^.

To investigate the effect of SS-31 on molecular mechanisms underlying the improvement of mitochondrial functions in BTHS, we isolated cardiac mitochondria from Taz^KD^ mice after administration of SS-31 (3 mg/kg/day) for 10 weeks. Lipidomics analysis by MALDI-TOF/MS was performed to check for the typical lipid alterations in Taz^KD^ mitochondria and to compare the lipid profiles of mitochondrial membranes in the presence and absence of SS-31 treatment.

We show that treatment with SS-31 does not affect the activity of individual enzymes of the OXPHOS system but leads to an improvement in mitochondrial respiratory capacity associated with an increase in supercomplex organization, without modifying the MLCL/CL ratio.

## Results

### Tafazzin-knockdown mice

Male Taz^KD^ and wild-type (WT) mice were studied at 4–6 months of age when mitochondria and CL abnormalities are well established, but the animals do not exhibit cardiac dysfunction^38–40^. At the time of sacrifice, the mice’s body weights were significantly lower in Taz^KD^ (27.6 ± 1.8 g) compared to the non-transgenic littermate controls (35.0 ± 3.6 g; *p* < 0.0001), while transgenic mice had normal heart weight (Table 1).

**Table 1:** Heart and body weights of mice.

	Body (g)	*p*-value	Heart (mg)	p-value	Heart/body (mg/g)	*p*-value
WT	35.0 (± 3.6)	< 0.0001	204.0 (± 48.3)	0.69	6.4 (± 0.6)	0.24
Taz^KD^	27.6 (± 1.8)		191.3 (± 40.5)		7.2 (± 1.1)	
Taz^KD^ + saline	29.2 (± 2.5)	0.42	183.3 (± 6.9)	0.89	6.3 (± 0.3)	0.42
Taz^KD^ + SS-31	28.0 (± 1.4)		184.3 (± 11.9)		6.6 (± 0.1)	

All values are means ± SD (WT and Taz^KD^: n = 9/group; Taz^KD^ + saline and Taz^KD^ + SS-31: n = 4/group). *p*-values were assessed by Student’s t-test.

Protein expression of tafazzin in cardiac muscle was quantified by densitometric analysis and showed an approximately 80% reduction in Taz^KD^ mice compared with doxycycline (dox)-fed WT animals (see Fig. S1). As expected, the addition of dox to the rodent diet markedly reduced the content of tafazzin protein in Taz^KD^ mitochondria. To investigate the effect of SS-31 on the BTHS animal model, 4-month-old male Taz^KD^ mice were treated with the drug (3 mg/kg) or saline, by subcutaneous injections once daily for a period of 10 weeks.

### Comparative lipid analysis by MALDI-TOF/MS of cardiac mitochondria isolated from WT and Taz^KD^ mice

To analyse the main mitochondrial lipid species, we acquired the MALDI mass spectra of the total lipid extracts obtained from isolated cardiac mitochondria in both negative and positive ion modes. Table 2 collects the main signals detected in the mass spectra attributable to the negative [M − H]^−^ and positive [M + H]^+^ molecular ions of mitochondrial lipids.

**Table 2:** Lipid assignments of *m/z* values detected in negative and positive ion mode MALDI-TOF mass spectra of heart mitochondria from WT and Taz^KD^ mice.

*m/z* value	[M − H]^−^	[M + H]^+^
435.2	LPA (18:1)	
496.3		LPC (16:0)
520.3		LPC (18:2)
524.3		LPC (18:0)
544.4		LPC (20:4)
568.3		LPC (22:6)
571.2	LPI (16:0)	
673.4	PA (34:1)	
695.4	PA (36:4)	
699.4	PA (36:0)	
723.5	PA (38:4)	
734.5		PC (32:0)
738.4	PE (36:4)	
742.5	PE (36:2)	
747.4	PG (34:1)	
758.5		PC (34:2)
760.5		PC (34:1)
762.4	PE (38:6)/PS (34:1)	
766.4	PE (38:4)	
774.5	PE (38:0)/PlsS (p-36:0) / PlsE (p-40:6)	
782.5		PC (36:4)
784.5		PC (36:3)
786.5		PC (36:2)
788.6	PS (36:1)	
790.4	PE (40:6)	
806.5		PC (38:6)
810.5		PC (38:4)
812.4	PS (38:3)	
834.5	PS (40:6)	
830.6		PC (40:8)
834.6		PC (40:6)
861.4	PI (36:2)	
885.4	PI (38:4)	
925.6	DLCL (36:3)	
927.6	DLCL (36:2)	
953.6	DLCL (38:3)	
1165.8	MLCL (52:2)	
1185.8	MLCL (54:6)	
1189.8	MLCL (54:4)	
1215.7	MLCL (56:5)	
1404.0	CL (68:2)	
1428.0	CL (70:4)	
1448.0	CL (72:8)	
1450.0	CL (72:7)	
1474.0	CL (74:9)	
1476.0	CL (74:8)	
1496.0	CL (76:12)	

The numbers (a:b) define the total length (as carbon numbers) and number of double bonds of acyl chains, respectively.

**Figure 1:**
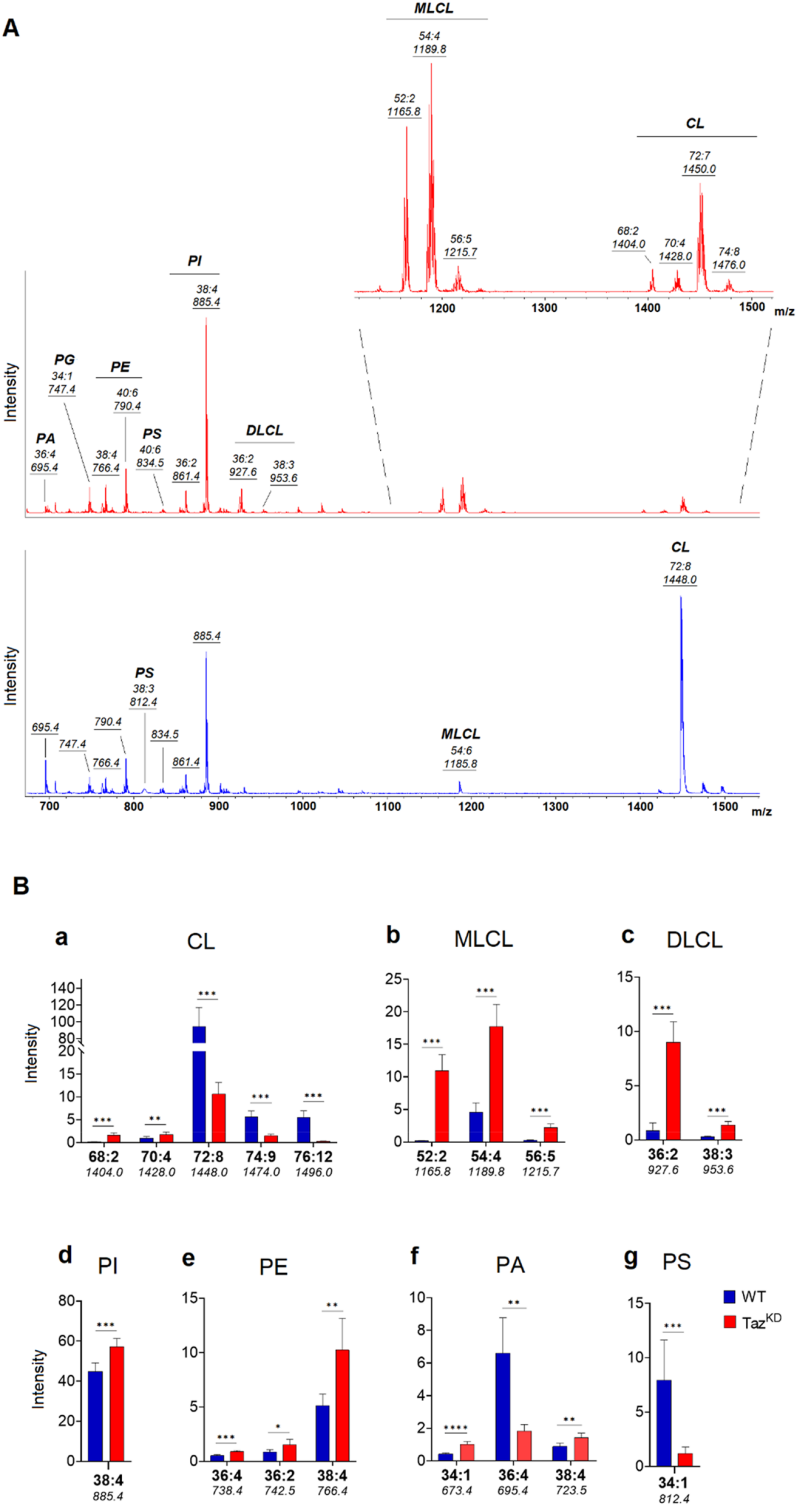
Negative ion mode MALDI-TOF/MS lipid profiles of Taz^KD^ (red) and WT (blue) cardiac mitochondria. Mitochondria were isolated from 4-month-old mice and representative lipid mass spectra are shown in (**A**). Lipid assignments for main signals are indicated. The enlargement of the range *m/z* 1100 to 1500 of the mass spectrum of Taz^KD^ is also shown. The histograms in (**B**) show the significant differences in intensity between the lipid peaks in the two series of (−) MALDI mass spectra. Data are reported as the average value of intensity ± SD. A *p*-value < 0.05 was set as the threshold for significant differences between the two series of mass spectra (*p < 0.05; **p < 0.01; ***p < 0.001). The numbers under each histogram indicate the detected MALDI *m/z* peaks.

Figure 1A shows the comparison between the representative lipid profiles of mitochondria isolated from WT (lower panel) and Taz^KD^ (upper panel) mice obtained by negative ion mode MALDI-TOF/MS. Both mass spectra show main peaks corresponding to various species of phospholipids, i.e. phosphatidylinositol (PI 36:2 and 38:4) and PE (38:4 and 40:6) species; minor peaks are attributable to phosphatidylglycerol (PG 34:1) and phosphatidic acid (PA 36:4) species. Additional signals detected in the *m/z* 900–1100 range of the mass spectra could be compatible with glycosphingolipid species. As expected, the most important differences between the two MALDI lipid profiles are in the higher mass range, where the signals diagnostic for MLCL and CL are detected (see Fig. 1A, inset of the upper panel). The CL fingerprinting of mitochondria from WT mice shows a major peak at *m/z* 1448.0 corresponding to tetralinoleoyl CL (CL 72:8), the main CLm form in cardiac mitochondria^15,28^. Because of the loss of function of *TAFAZZIN* in CL remodeling, the MALDI mass spectrum of mitochondria from Taz^KD^ animals contains a cluster of peaks (at *m/z* 1165.8, 1189.8, and 1215.7) of interest diagnostics for the presence of MLCL species (MLCL 52:2, 52:4, 56:5, respectively). A very small signal corresponding to MLCL 54:6 having three molecules of linoleic acid can also be detected in the mass spectrum of WT mitochondria at *m/z* 1185.8 (Fig. 1A, lower panel). As for the signals attributable to CL species in Taz^KD^ mitochondria, the major peak is detected at *m/z* 1450.0 and corresponds to another CLm form, CL 72:7. The MALDI signals at *m/z* 1404.0, 1428.0, and 1476.0 are diagnostic for the presence of an altered acyl CLs composition (CLi species) in the heart, which have a higher content of monounsaturated acyl chains (CL 68:2, 70:4, and 74:8, respectively) (see Fig. 1A, upper panel). Indeed, MS comparative analysis confirmed a strong reduction of the signal corresponding to CL 72:8 at *m/z* 1448.0 in Taz^KD^ mitochondria compared with WT mitochondria, with a contextual accumulation of three MLCL species [see Fig. 1B (a,b)]. Furthermore, two peaks at *m/z* 927.6 and 953.6 corresponding to dilysocardiolipin (DLCL) species (DLCL 36:2 and 38:3) are visible in the mitochondrial lipid profile of Taz^KD^, whereas they are almost absent in that of WT mice [Fig. 1A, inset and Fig. 1B(c)].

Interestingly, by comparing a series of replicates of WT and Taz^KD^ mass spectra, significant differences in the intensity of MALDI signals corresponding to other mitochondrial glycerophospholipids were also detected, indicating more widespread effects of tafazzin deficiency on membrane lipid composition than CL (and MLCL) alone [see Fig. 1B (d–g)]. More specifically, the major signal at *m/z* 885.5 attributable to PI 38:4 and the minor peaks (at *m/z* 738.4, 742.5, and 766.4) assigned to various PE species were significantly higher in the Taz^KD^ lipid profile than in WT mice [Fig. 1B (d,e)]. Other noteworthy phospholipid changes were relative to the MALDI signals of PA 36:4, identified by the peak at *m/z* 695.4, and PS 34:1 at *m/z* 812.4, whose intensity decreased significantly in the Taz^KD^ lipid profile compared with control mice [Fig. 1B (f,g)]. In contrast, peaks at *m/z* 673.4 and 723.5, corresponding to PA 34:1 and 38:4, respectively, were significantly higher in the Taz^KD^ lipid profile [Fig. 1B (f)]. Various signals attributable to other acidic phospholipid species, such as PG, lysophosphatidylinositol (LPI), and lysophosphatidic acid (LPA), whose MALDI signals in negative ion mode did not change significantly when the two lipid patterns were compared, are collected in Table 2.

**Figure 2:**
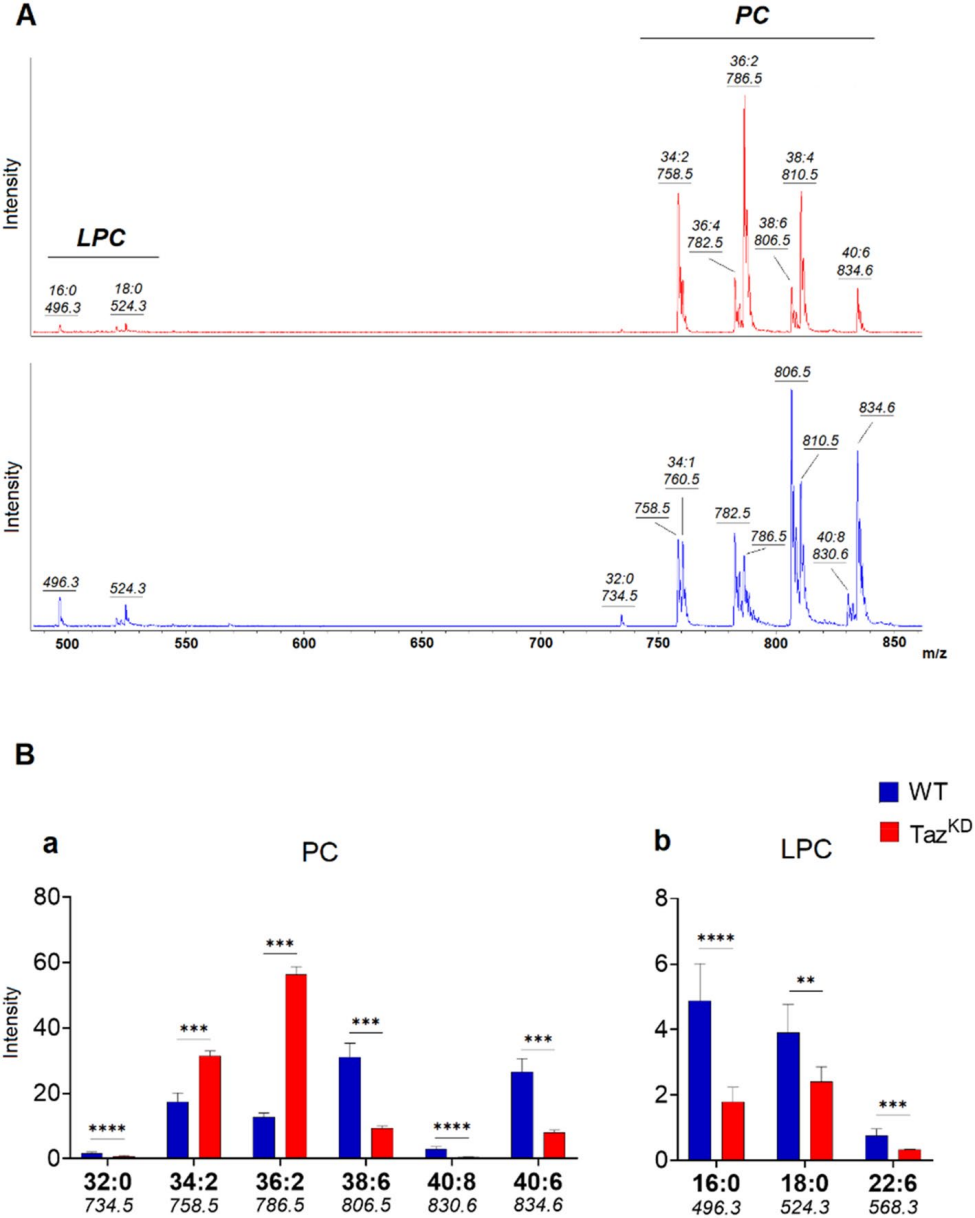
Positive ion mode MALDI-TOF/MS total lipid profiles of Taz^KD^ (red) and WT (blue) cardiac mitochondria (**A**). Mitochondria were isolated from 4-month-old mice and representative lipid mass spectra are shown in (**A**). Lipid assignments for main signals are indicated. The histograms (**B**) show the significant differences in intensity between the lipid peaks in the two series of (+) mass spectra. Data are reported as the average value of intensity ± SD. A p-value < 0.05 was set as the threshold for significant differences between the series of mass spectra (*p < 0.05; **p < 0.01; ***p < 0.001, ****p < 0.0001). The numbers under each histogram indicate the detected MALDI *m/z* peaks.

To detect zwitterionic phospholipids, we also performed positive ion mode MALDI-TOF/MS analysis of total lipid extracts from cardiac mitochondria of WT (Fig. 2A, lower panel) and Taz^KD^ (Fig. 2A, upper panel) mice (see also Table 2). Significant differences in the intensity of MALDI signals detected between the two lipid profiles are shown as histograms in Fig. 2B. Both positive ion MALDI mass spectra are dominated by several signals identified as PC species and have smaller peaks diagnostic for lysophosphatidylcholine (LPC) species in the lower mass range. The most evident difference between the two lipid profiles is the change in the main signal corresponding to PC 38:6 (at *m/z* 806.5) in WT mitochondria, whereas the peak attributable to PC 36:2 (at *m/z* 786.5) is most intense in the Taz^KD^ profile (Fig. 2A).

The intensities of the peaks at *m/z* 758.5 and 786.5, assigned to the two PC species 34:2 and 36:2, respectively, containing less unsaturated fatty acids, were significantly higher in Taz^KD^ mice compared with the control animals [Fig. 2B (a)]. These elevated PC levels might be related to tafazzin deficiency, which also correlates with lower signals at *m/z* 496.3, 524.3, and 568.3 diagnostics for the presence of LPC species (LPC 16:0, 18:0, and 22:6, respectively) in Taz^KD^ mice [Fig. 2B (b)]. This condition might be due to the reduced activity of transacylation between CL and PCs in BTHS animals. On the other hand, other PC species, identified by peaks at *m/z* 734.5, 806.5, 830.5, and 834.6, present lower levels in Taz^KD^ mice compared with WT littermates [Fig. 2B (a)].

Overall, our MALDI results confirmed that tafazzin deficiency strongly affects the lipid composition of the cardiac mitochondrial membrane in Taz^KD^ mice, i.e. both the CL fingerprint and the content of other phospholipids that are critical for membrane structure and function.

To test the effect of SS-31 as a pharmacological agent to improve mitochondrial bioenergetics in the BTHS animal model, we first analysed the lipids of cardiac mitochondria from treated Taz^KD^ mice by MALDI-TOF/MS. Comparative lipid analyses of mitochondria from Taz^KD^ mice treated with SS-31 or saline revealed neither qualitative nor quantitative differences in phospholipid composition (see Fig. S2). No significant differences were observed in the intensity of MALDI peaks in both negative and positive ion mode spectra (not shown).

**Figure 3:**
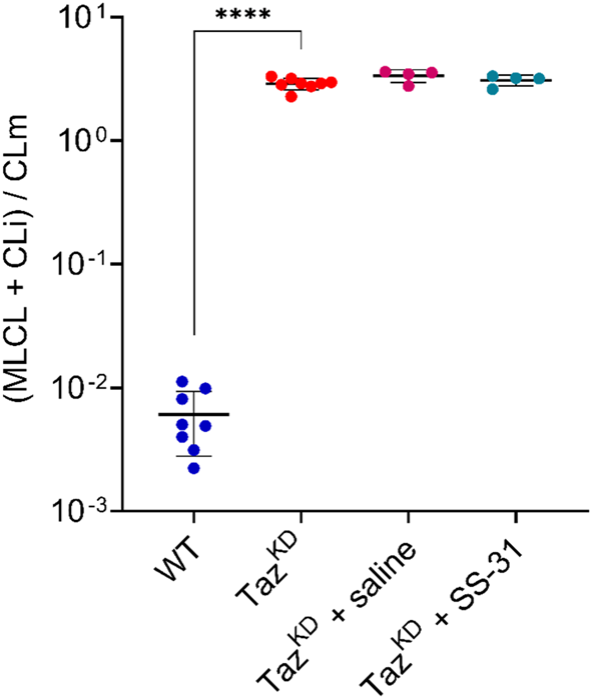
(MLCL + CLi)/CLm ratios calculated by MALDI-TOF/MS analysis of mitochondria. Mitochondrial lipids were analysed by (−) MALDI analysis (in triplicate for each sample) and ratios were calculated as described in “Methods”. The scatter plot shows the measurements of all mitochondrial samples for each group of mice (8 WT, 8 Taz^KD^, 4 Taz^KD^ + saline, and 4 Taz^KD^ + SS-31) and the average values with the error bars indicating SD (****p < 0.0001). The *y*-axis shows a logarithmic scale.

As regards the CL fingerprinting alteration in Taz^KD^ mitochondria, we also calculated the MLCL/CL ratio as a lipid marker for the SS-31 effect. Figure 3 summarizes the (MLCL + CLi)/CLm ratios calculated using MALDI-TOF/MS as previously reported^25,26^ in mitochondria isolated from four different animal groups: WT and Taz^KD^ (4-months-old mice) and Taz^KD^ treated with SS-31 or saline (6 and a half months old mice). As expected, the (MLCL + CLi)/CLm ratio calculated in normal mitochondria was significantly lower than that in mitochondria isolated from tafazzin-deficient animals. While in vivo treatment of Taz^KD^ mice with SS-31 for a relatively long period did not significantly affect the calculated ratio, the lipid ratio also did not change between Taz^KD^ animals of different ages (Fig. 3).

### SS-31 ameliorates mitochondrial respiratory capacity and promotes supercomplex formation in Taz^KD^ mice

To shed light on the effect that SS-31 treatment might elicit on Taz^KD^ mitochondrial dysfunction, we examined the mitochondrial respiration rate and supercomplex organization in cardiac mitochondria isolated from the different groups of mice.

**Figure 4:**
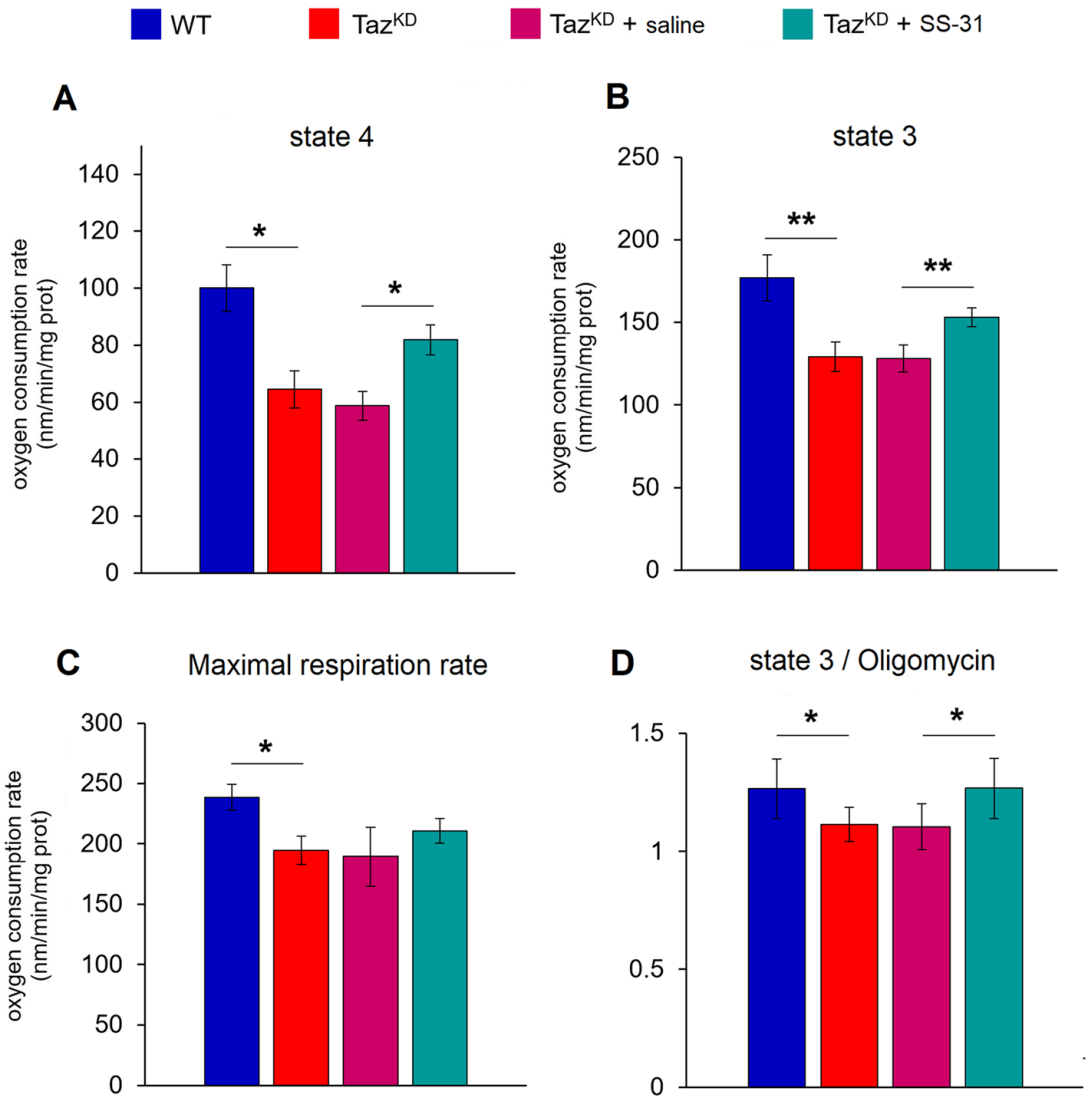
Cardiac mitochondria respiratory rates and effect of SS-31. The oxygen consumption rate of non-phosphorylating (state 4) (**A**) and phosphorylating (state 3) (**B**) with succinate as respiratory substrate. Full uncoupling of the succinate-dependent respiration rate was assessed in the presence of 0.25 μM CCCP (**C**). The respiratory control ratio (RCR) was calculated by the state 3/post-oligomycin respiration ratio (**D**). Histograms display the mean values ± SD (n = 4/group). Statistically significant differences are indicated: Student’s t-test (**p < 0.01, *p < 0.05).

First, oxygen consumption analysis of isolated mitochondria revealed a significant decrease in both non-phosphorylating (state 4) and phosphorylating (state 3) succinate-supporting respiration in Taz^KD^ mice compared with WT, which was accompanied by a decrease in maximal respiration rate (CCCP) (Fig. 4A–C). Treatment of Taz^KD^ mice with SS-31 resulted in significant improvement of mitochondrial respiration in both states 3 and 4 compared with vehicles-treated Taz^KD^ mice (Fig. 4A,B). Although SS-31 treatment tended to increase maximal respiration rate, no statistically significant effect was observed (Fig. 4C). Moreover, we found that the ratio of state 3/post-oligomycin respiration was lower in Taz^KD^ mice than in WT, but it was restored in SS-31-treated mice compared with saline-treated mice (Fig. 4D).

**Figure 5:**
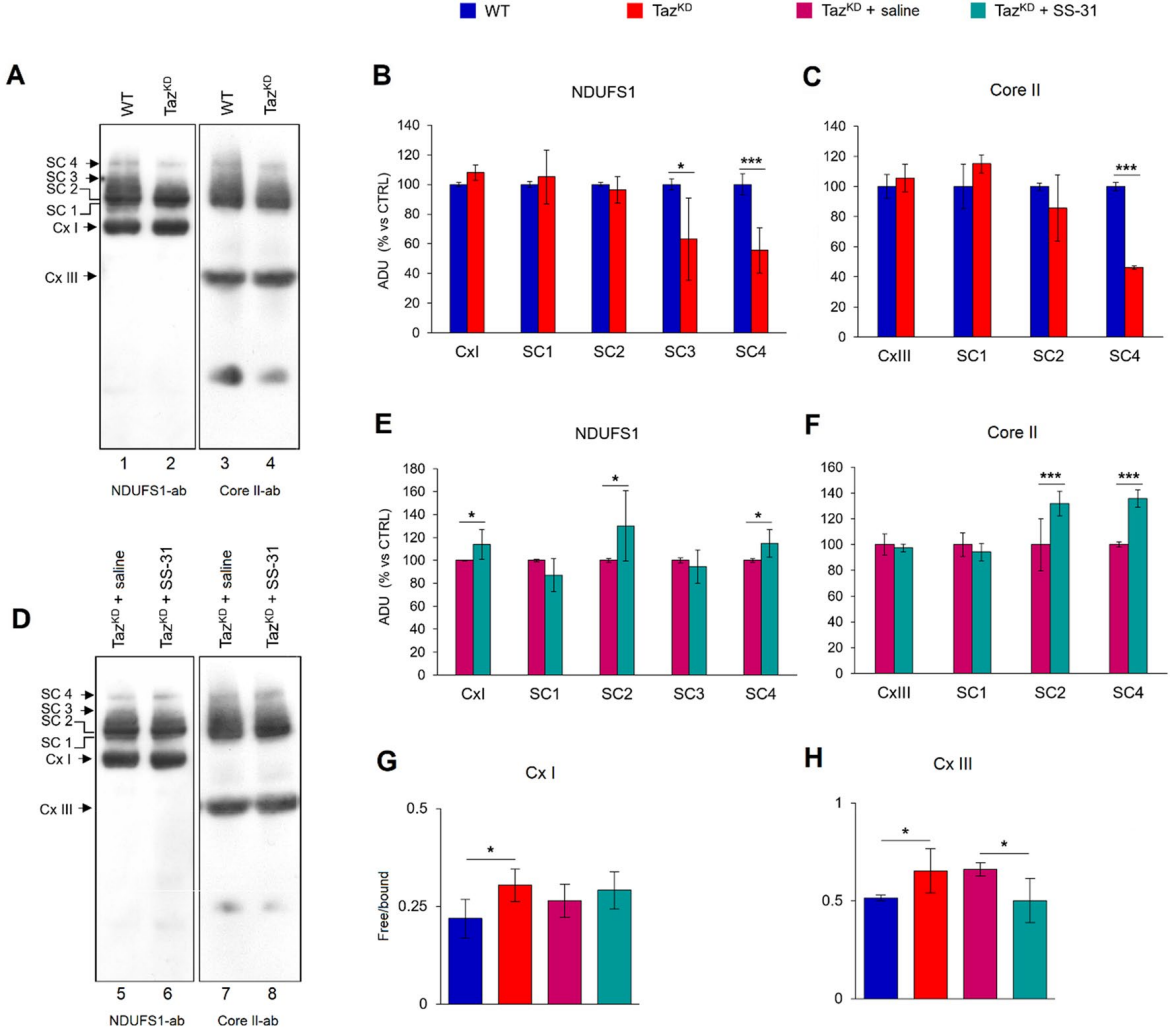
Respiratory chain supercomplex organization analysis. Isolated cardiac mitochondria were separated by BN-PAGE, transferred onto PVDF membrane, and subjected to western blotting analysis with specific antibodies, as indicated. Supercomplexes (SC4, SC3, SC2, and SC1) containing complex I and complex III in various amounts are shown. Complex I (Cx I) and complex III (Cx III) are also identified as monomers (**A**, **D**). Protein levels estimated by band densitometry (**B**, **C**, **E**, **F**) in Taz^KD^ and Taz^KD^ + SS-31 are expressed as percentages vs WT and Taz^KD^ + saline, respectively. Histograms **G** and **H **display the percentage of ADU of immune-revealed bands in free complexes and in supercomplexes in each lane. All histograms show the mean values ± SD (n = 4/group). Statistically significant differences are indicated: Student’s t-test (**p < 0.01, *p < 0.05).

The efficiency and capacity of mitochondrial electron transport could be improved by respiratory chain organization into large complexes called respiratory chain supercomplexes (SCs) ^41,42^. Figure 5 shows the results obtained by blue native electrophoresis (BN-PAGE) of digitonin-solubilized isolated cardiac mitochondria, followed by immunoblotting analysis with specific antibodies against a subunit of complex I (Cx I) and a subunit of complex III (Cx III). In WT mitochondria, immunoblotting of NDUFS1, a subunit of complex I, revealed the free complex I and higher orders of assembly of complex I with higher molecular weights of which the more discrete bands have been referred as SC1, SC2, SC3 and SC4 (Fig. 5A, lanes 1–2). The antibody against the Core II, subunit of complex III, revealed the free complex III and bands with the same molecular weight as that immuno-revealed with the NDUFS1 antibody, indicating the presence of the complex III in SC2, SC3, and SC4 (containing at least complex I and complex III) (Fig. 5A, lanes 3–4). Densitometric analysis of the NDUFS1-immuno-revealed bands showed that SC3 and SC4 decreased in Taz^KD^ mitochondria compared with WT (Fig. 5B). This is also confirmed by densitometric analysis of the Core II-immuno-reveled bands (Fig. 5C). No difference was observed in free complex I and III between the two groups of mice (Fig. 5B,C). Treatment of Taz^KD^ mice with SS-31 increased SC2 and SC4 levels compared with vehicles-treated mice (Fig. 5D–F). In addition, a significant increase in the amount of free complex I was observed in the treated Taz^KD^ mice (Fig. 5E), whereas complex III did not appeared to be affected by treatment with SS-31 (Fig. 5F). Moreover, the specific distribution of complex I and complex III, free or bound in the SCs, changed in Taz^KD^ with respect to WT mice. Indeed, the ratio between free complexes and SCs containing complex I or complex III increased in Taz^KD^ mice, and the treatment with SS-31 decreased the ratio only for complex III and not for complex I (Fig. 5G,H). This suggested a higher percentage of free complex I and complex III in Taz^KD^ mitochondria. Instead, treatment with SS-31 rescued only the percentage of free complex III, without affecting that of complex I.

## Discussion

SS-31, the pharmacological agent used in this study to treat Taz^KD^ mice, belongs to the Szeto-Schiller (SS) peptide family ^43^. It is an aromatic-cationic and water-soluble small peptide, which crosses the outer mitochondrial membrane and localizes to the inner membrane, where it binds to CL (as well as to MLCL if present) via electrostatic and hydrophobic interactions^36,44,45^. Its effects have been studied extensively by many independent investigators in aging and various disease models, and it has been found to restore cellular bioenergetics by improving mitochondrial structure and function, increasing energy production, and reducing ROS generation to near-normal levels^36,43,44,46^.

With regard to the treatment of BTHS cardiomyopathy, SS-31 has been shown to rapidly improve both mitochondrial bioenergetics and morphology in induced pluripotent stem cells (iPSCs) from BTHS patients^27^. The beneficial effects of SS-31 have been demonstrated in animal models of experimental left ventricular dysfunction and heart failure^47–49^. Chronic treatment with SS-31 has also been reported to improve cardiac function and to positively remodel the CL composition in rodent and canine models^47,50^. However, it is still unclear whether the CL post-treatment remodeling is a cause or a consequence of the mechanism of action of SS-31. In contrast, few data sets on SS-31 have been used in non-clinical BTHS models. As far as we know, the effects of long-term treatment with SS-31 on cardiac mitochondrial dysfunction in mouse models of BTHS have not been reported in the scientific literature. There is also a lack of data on the analysis of the different CL species and their fatty acyl chain composition after administration of SS-31 to animal models. Therefore, the aim of this study was to treat Taz^KD^ mice with SS-31 to evaluate the heart for changes in phospholipid profiles as well as to explore at the molecular level any associated effects on mitochondria.

As expected, lipidomic analysis of cardiac mitochondria by MALDI-TOF/MS revealed about 90% decrease in the content of the major CLm species (CL 72:8) in Taz^KD^ mitochondria compared with WT, with a dramatic accumulation of MLCL species and an alteration in the molecular species composition of CLi (CL 68:2 and 70:4) due to tafazzin deficiency. Furthermore, we detected increased levels of two different molecular DLCL species, possible degradation products of MLCL, in Taz^KD^ lipid profiles. Regarding the content of other lysophospholipids, we found that the amount of the main LPC species containing either unsaturated or saturated fatty acids was significantly lower in Taz^KD^ mice than in control animals. These data are consistent with the decreased activity of transacylation between PCs and CL in BTHS animals.

Other mitochondrial phospholipid levels were also altered, highlighting the wider role of tafazzin in affecting other lipids content than CL (and MLCL) in the mitochondrial membrane. When Taz^KD^ and WT mitochondria were compared, the content of the main PI and PE species was found to be significantly higher in tafazzin-deficient mice, while the content of PS was lower. The most evident compositional change was observed in acidic phospholipid 38:4 species likely containing polyunsaturated fatty acids (PUFA), such as PI, PE, and PA, whose content significantly increased in Taz^KD^ mitochondria.

As for the physiological role of PC in phospholipid metabolism, it has been well established that it (together with PE) acts as acyl donor for tafazzin-mediated CL remodeling^9,51^. The most important change in PC composition was an increase in the content of PC probably containing linoleic acid (PC 34:2 and 36:2), and a corresponding decrease in PC species containing long-chain PUFA in Taz^KD^ mitochondrial membrane, as previously reported in^31^. This imbalance of molecular species driven by tafazzin deficiency to incorporate linoleic acid into CL dramatically alters both mitochondrial membrane biophysical properties and cellular signaling processes that use these PUFA as signaling molecules in the myocardium of BTHS animals.

These important phospholipid alterations were generally associated with impaired mitochondrial respiratory chain enzymes in Taz^KD^ mice^27,40,52^. Here, we found that Taz^KD^ cardiac mitochondria exhibited reduced respiratory rates in state 3 and 4 with a reduction in maximal respiratory capacity. In addition, analysis by BN-PAGE showed that tafazzin-deficient mitochondria had lower levels of complex I and complex III in respiratory supercomplexes than normal mitochondria, accompanied by an increase of percentage of free complexes I and III. Our data confirm the well-known importance of the content and composition of CL in stabilizing the mitochondrial respiratory chain supercomplexes to maintain normal mitochondrial function and optimize the oxidative phosphorylation system^53^. In a recent report using the same BTHS model, tafazzin deficiency was shown to selectively impair pyruvate and fatty acid oxidative metabolism in cardiac mitochondria, but to cause up-regulation of glutamate oxidation by the malate-aspartate shuttle as a compensatory effect^52^.

In vivo administration of SS-31 to our BTHS mouse model did not significantly alter mitochondrial phospholipid composition. In addition, the MLCL/CL ratio in Taz^KD^ cardiac mitochondria, which was used for therapeutic monitoring of drug treatment, was not affected after 10 weeks of treatment. Therefore, we can conclude that SS-31 administration to Taz^KD^ mice under our experimental conditions does not affect phospholipid profiles, in particular, the CL fingerprint of cardiac mitochondria. On the other hand, measurement of the MLCL/CL ratio can be expected to have a more direct correlation to the efficacy of enzyme replacement or gene therapy but not of a CL-targeted agent.

Moreover, we found that treatment of Taz^KD^ mice with SS-31 significantly restored the reduction in respiration rates in both succinate-dependent states 3 and 4 indicating an amelioration of mitochondrial function. Since it has been reported that SS-31 can increase the activity of the complexes II and III by inducing the biogenesis of Fe-S clusters^54^, the improvement in mitochondrial respiration rates observed here could be ascribed to the increase in the activities of the respiratory chain complexes. However, under our experimental conditions, we feel to exclude a SS-31-dependent increase of complex II activity, because the treatment of Taz^KD^ mice did not increase the succinate-dependent maximal respiration rate (in the presence of CCCP). In addition, the in-gel activity assay suggested that SS-31 did not affect the activity of complex IV as well as the activities of complexes I and V (Fig. S3 and Supplementary Table S1). All these data indicate that SS-31 restores mitochondrial function without affecting the activities of the free respiratory chain complexes.

Nevertheless, an increase of state 3/post-oligomycin respiration ratio was observed in SS-31 treated samples, with respect to saline-treated samples. The state 3/post-oligomycin respiration ratio allows calculation of the respiratory control ratio (RCR), providing an indication of the efficiency of the OXPHOS system, which means the mitochondrial respiratory chain efficiency coupled with ATP synthesis^55^. Thus, our results showed a decrease in mitochondrial efficiency of Taz^KD^ mice compared with WT which was rescued by SS-31 treatment. Since it has been proposed that supercomplexes play a role in increasing the respiratory chain efficiency/capacity^56–58^, we analysed supercomplexes organization by BN-PAGE. The composition of supercomplexes may vary depending on cell type and cell energy requirements, thus a ‘plasticity model’ was hypothesized^59^. Respiratory active supercomplexes, containing complexes I/II/III/IV, and I/III/IV were found^59–61^. Accordingly, we found deregulation of mitochondrial supercomplex organization in Taz^KD^ mice with respect to WT, and, interestingly, treatment with SS-31 was associated with an increase in respiratory supercomplexes formation. This agrees with the increased efficiency indicated by the rescue of the state 3/post-oligomycin respiration ratio. Moreover, treatment with SS-31 also resulted in a decrease of percentage of free complex III with respect to complex III in supercomplexes, but not that of complex I. This lack of effect on the percentage of free complex I could be attributed to the effect of supercomplexes on the promotion of assembly and stability of complex I^62^. Together the data suggested that the SS-31-dependent amelioration of mitochondrial functions (state 4, state 3 and state 3/post-oligomycin ratio) was associated with the increased supercomplexes formation.

Our results are consistent with the well-established role of CL in stabilizing respiratory supercomplexes^63^ and with the protective effects of SS-31 on mitochondria. Indeed, this peptide can increase the electron flux in the respiratory chain, the oxygen consumption, the ATP production, and the P/O coupling through interaction with CL on the mitochondrial inner membrane^64,65^. Mechanistically, it has been proposed that binding of SS-31 to the key mitochondrial phospholipid maintains cristae structure and induces inner membrane curvatures that promote the assembly of respiratory complexes into supercomplexes to facilitate electron transfer^66^.

On the other hand, encouraging clinical results on the use of SS-31 in the treatment of BTHS patients support the potential use of the peptide for the management of this rare disease. The efficiency of SS-31 in BTHS patients was recently evaluated in a randomized phase 2/3 clinical trial followed by an open-label extension (TAZPOWER trial). Significant improvements in the 6-min walk test and in the BTHS symptom assessment scale were observed after 36 weeks of SS-31 exposure^67^. Furthermore, the findings showed an improvement in knee extensor muscle strength and in some cardiac parameters in the treated patients, such as a significant increase in the average cardiac stroke volume from the trial baseline to week 36 of the open-label extension^67^. Regarding the value of MLCL/CL as a temporal measure of clinical condition, no change has been reported in subjects treated with SS-31^67^, in agreement with our present results.

In conclusion, we report for the first time that SS-31 can improve cardiac mitochondrial function without altering CL composition in a BTHS mouse model. Our results, showing no changes in the levels of CL (and MLCL), are consistent with the hypothesis that chronic treatment with SS-31 exerts its beneficial effects by affecting the mitochondrial electron transport chain function (i.e*.* by improving the stabilization of CL-protein interactions) rather than by directly affecting CL composition.

Nevertheless, we cannot exclude the possibility that drug treatment may not exert its chronic effect on the CL composition of Taz^KD^ mitochondria because BTHS mice lack remodeling capabilities.

Further studies will be necessary to understand whether the ultrastructure of Taz^KD^ mitochondrial membrane is affected by administration of the peptide and to elucidate the precise mechanism of action on mitochondrial bioenergetics in the heart.

## Methods

### Animal model and SS-31 administration

Taz^KD^ mice used in this study were generated by mating male mice [B6.Cg-Gt(ROSA)26Sor < tm37(H1/tetO-RNAi:Taz)Arte > /ZkhuJ; The Jackson Laboratory, Bar Harbor, ME] which are transgenic for dox—inducible *TAFAZZIN* specific short hairpin RNA (shRNA) with female C57BL/6J mice (The Jackson Laboratory, Bar Harbor, ME) as previously described^38,39^. The use of shRNA Taz^KD^ mice overcomes the very high embryonic lethality of complete genetic ablation of *TAFAZZIN* ^38,39^. *TAFAZZIN* knockdown was induced in utero and maintained postnatally by TD.01306 chow (Envigo, IN, USA) supplemented with 625 mg/kg dox. The different dox administration strategies and concentrations resulted in variable cardiac phenotypes and results^33,34^. Dox was administered to female mice continuously to induce the embryonic transgene throughout gestation. Because males carry the transgene as heterozygotes, mating pairs were fed regular chow to reduce potential sterility, which is a known adverse effect in a *Drosophila* model of tafazzin deficiency^68^. Dox was re-administered to females the day after mating (day of plug). Offspring were genotyped by PCR analysis of genomic tail DNA for the 381-bp *TAFAZZIN* shRNA gene product (see Fig. S4), as previously described^38^. Both Taz^KD^ and WT mice were fed the dox diet until the day of sacrifice to induce shRNA expression and control for unintended effects of dox in WT animals. Only male mice were used for the study. SS-31 (New England Peptide Inc, USA) or normal saline as a vehicle was administered to 4-month-old Taz^KD^ mice with a subcutaneous dose of 3 mg/kg/day for 10 weeks. The SS-31 dose was chosen based on previous optimizations in mouse models of various diseases^43,69,70^.

Taz^KD^ and WT littermates were sacrificed at 4 months of age, whereas mice that received the SS-31 treatment (or saline) were 10 weeks older (about 6 and a half months of age). At this age, the mice exhibit significant lipid and mitochondrial abnormalities but no cardiac dysfunction^38,39^.

The study was approved by the Ethics Committee of the University of Bari Aldo Moro and by the Italian Ministry of Health following internationally accepted guidelines for animal care (Approval number 162/2020-PR on 5 March 2020). Animals were kept under a 12-h dark-to-light cycle, constant room temperature and humidity (22 ± 2 °C, 75%), with food and water ad libitum. Mice were deeply anesthetized with ketamine (100 mg/kg, intraperitoneally) before being sacrificed by cervical dislocation and removal of the heart.

### Heart mitochondria isolation

Mitochondria were isolated from fresh adult mouse cardiac muscle, using a differential centrifugation method previously described^71,72^. Hearts were quickly excised and placed in ice-cold 0.9% NaCl solution. The blood was removed by washing and the connective tissue was cut off, then the hearts were minced in 2 mL of ice-cold mitochondria isolation buffer (MIB) consisting of 250 mM sucrose, 10 mM HEPES (pH 7.4), 5 mM EGTA, and 0.25 mM phenylmethylsulfonyl fluoride (PMSF). The minced hearts were gently homogenized using a Dounce glass-Teflon homogenizer. After centrifugation at 800×*g* for 10 min at 4 °C, 200 μl of supernatant was collected and stored rapidly at − 80 °C; homogenates were used for western blotting, as described below. Mitochondria were sedimented by centrifugation of the remaining supernatant at 10,000×*g* for 10 min at 4 °C. The resulting pellet was washed once with MIB and finally resuspended in a small volume of MIB. Total protein concentration was determined by the Bradford protein assay.

### Mitochondrial respiratory rates

Respiratory activity of freshly isolated cardiac mitochondria was measured polarographically with a Clark-type oxygen electrode in a water-jacketed chamber magnetically stirred (Hansatech Instruments) at 37 °C as described in^73^. Briefly, mitochondria were suspended at a final protein concentration of 0.25 mg/ml in 75 mM sucrose, 50 mM KCl, 30 mM Tris, 2 mM KH_2_PO_4_, 2 mM MgCl_2_, and 10 μM EGTA. Respiration was started by adding 10 mM succinate in the presence of 1 μg/ml rotenone to inhibit complex I (state 4). State 3 respiration was induced by the addition of 1 mM ADP. Other mitochondrial respiratory chain substrates were added at the following concentrations: oligomycin (1 µg/ml), and carbonyl cyanide m-chlorophenyl hydrazone (CCCP 0.25 µM).

### Electrophoretic procedures and western blotting

Heart homogenates (20 µg protein) were mixed with Laemmli Sample Buffer 4x (Bio-Rad, California, USA), and dithiothreitol (DTT). After incubation for 10 min at 37 °C, samples were loaded into 10% polyacrylamide gels (BioRad TGX FastCast Acrylamide Kit, #1610173), electrophoresed and then transferred to a nitrocellulose membrane. The membranes were blocked in 5% non-fat dry milk in Tris-Buffered Saline (TBS) containing 20 mM Tris and 500 mM NaCl (pH-adjusted to 7.5) with 0.05% Tween-20 (Sigma-Aldrich, P1379) (TBS-T) for 1 h at 20 °C and then incubated with primary antibody diluted in 5% non-fat dry milk overnight at 4 °C with constant shaking. The following primary antibodies were used: anti-tafazzin (1:4000, Thermo Fisher Scientific, #703032), anti-β-Actin (1:8000, Cell Signaling Technology, #58169). After washing, membranes were incubated with anti-rabbit (1:5000) or -mouse (1:5000) HRP-conjugated secondary antibodies at 20 °C for 1 h. Reactive proteins were revealed using an enhanced chemiluminescence detection system (Clarity Western ECL Substrate, Bio-Rad, California, USA) and visualized on a Chemidoc Touch Imaging System (Bio-Rad, California, USA). Densitometric analysis was performed using Image Lab (Bio-Rad, California, USA).

### Blue native page and supercomplexes analysis

For respiratory chain supercomplexes analysis, 50 µg of mitochondrial proteins were suspended in Buffer A containing 750 mM aminocaproic acid, 50 mM Bis–Tris, 0.5 mM EDTA, pH 7.0, and treated with digitonin (6 g/g protein) for 20 min on ice. After incubation, samples were centrifuged at 20,000×*g* for 20 min at 4 °C. The supernatant was collected and Coomassie Brilliant Blue G-250 was added at a ratio of 1/20 (v/v). The proteins were loaded into BN-PAGE on a 3–13% gradient polyacrylamide gel, transferred to a PVDF membrane, and subjected to western blotting analysis with specific antibodies against the NDUFS1 subunit of complex I (1:200; Santa Cruz Biotechnology sc-99232) and the Core II subunit of complex III (1:1000, Invitrogen, 459220). Blots were incubated with HRP-conjugated anti-rabbit (1:5000) or -mouse (1:5000) secondary antibodies for 1 h at 4 °C. Protein bands were visualized using an ECL reagent. Image acquisition and densitometric analysis were performed using Image Lab (Bio-Rad, California, USA).

### Matrix-Assisted Laser Desorption Ionization-Time-Of-Flight/Mass Spectrometry (MALDI-TOF/MS)

MALDI-TOF mass spectra were acquired in the negative and positive ion modes on a Bruker Microflex LRF mass spectrometer (Bruker Daltonics, Bremen, Germany). The system used a pulsed nitrogen laser, emitting at 337 nm, the extraction voltage was 20 kV, and gated matrix suppression was applied to prevent detector saturation (up to 400 Th). For each mass spectrum, 2000 single laser shots (sum of 4 × 500) were averaged. The laser fluence was kept about 5% above the threshold to have a good signal-to-noise ratio. All spectra were acquired in a reflector mode (detection range: 400–2000 mass/charge, *m/z*) using the delayed pulsed extraction. Peak areas, spectral mass resolutions, and signal-to-noise ratios were determined using the software Flex Analysis 3.3 (Bruker Daltonics). A lipid mixture was spotted next to the sample, and external calibration was performed before each measurement, as previously described^74,75^.

The (MLCL + CLi)/CLm ratio was defined as the sum of the values of the mass spectrometric peak areas of the MLCL and CLi species on the value of the peak areas of the CLm species, detected in the same (−) MALDI mass spectrum of a given sample^25,26^. To calculate the (MLCL + CLi)/CLm ratio, only the first isotopologue of the following species was considered: MLCL 52:2 at *m/z* 1165.8 and MLCL 54:4 at *m/z* 1189.8; CLi 68:2 at *m/z* 1404.0 and CLi 70:4 at *m/z* 1428.0; CLm 72:8 at *m/z* 1448.0 and CLm 72:7 at *m/z* 1450.0. In addition, a correction for the overlapping between the M + 2 isotopologue of the CLm 72:8 and the monoisotopic peak of CLm 72:7 was introduced, as previously reported^25^.

### Preparation of samples for MALDI-TOF lipid analysis

Mitochondrial lipids were analysed using a rapid lipid extraction protocol suitable for small amounts of samples^25^. Briefly, the equivalent volume of 20 μg protein of the mitochondrial suspension was spun at 16,000×*g* for 30 s. The supernatant was discarded, and the remaining pellet was resuspended with 10 μl CHCl_3_. Then 10 μl matrix solution (10 mg/ml 9-aminoacridine (9-AA) in 2-propanol-acetonitrile, 60/40, v/v) was added to the pellet. The pellet was pipetted and dispersed repeatedly. After centrifugation, the supernatant was deposited (in triplicate) in droplets of 0.35 μl on the target (Micro Scout Plate, MSP 96 ground steel target) for MALDI-TOF/MS analysis after evaporation of the matrix.

### Statistical analysis

The significance of differences between data sets presented in western blotting and respiratory analysis was determined using Student’s t-test.

Series of MALDI mass spectra (three replicates) were averaged using the software ClinProTools 3.0 (Bruker Daltonics) to determine the area under the peaks. Significant differences between series of mass spectra were determined using Student’s t-test when the dependent variable was normally distributed and Wilcoxon/Kruskal–Wallis test when the distribution did not fit the normality assumption.

A *p*-value < 0.05 was considered statistically significant.

### Ethical approval

The study is approved by the Ethics Committee of the University of Bari Aldo Moro (OPBA) and by Italian Ministry of Health (Approval number 162/2020-PR on 5 March 2020) in accordance with internationally accepted guidelines for animal care.

## Supplementary Information

Supplementary Information.

## Data Availability

The datasets generated for this study will be made available on request to the corresponding author.
