# Supplementary material for: Beneficial effects of SS-31 peptide on cardiac mitochondrial dysfunction in tafazzin knockdown mice
Source: Sci Rep. 2022 Nov 18;12:19847. doi: 10.1038/s41598-022-24231-4 (PMC9674582; doi:10.1038/s41598-022-24231-4)
Supplement: Supplementary file 1 — Supplementary Information. [file 41598_2022_24231_MOESM1_ESM.pdf]

**Supplementary information**

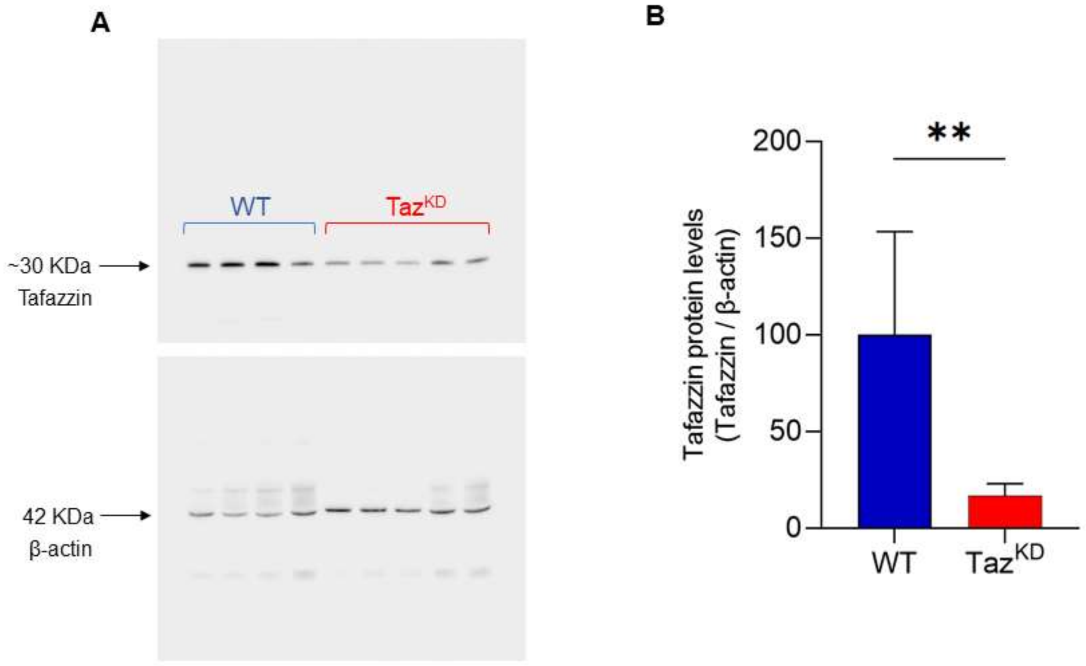


**<u>FIGURE S1. Tafazzin protein levels in WT and Taz$^{KD}$ knockdown mice.</u>** Representative (A) and graphical summary (B) immunoblot of Tafazzin protein detected in cardiac homogenates from WT (n=4) and Taz$^{KD}$ mice (n=5). Histogram shows the average of the percent arbitrary densitometric unit (ADU) values ± SD. Tafazzin levels were normalised by β-actin.

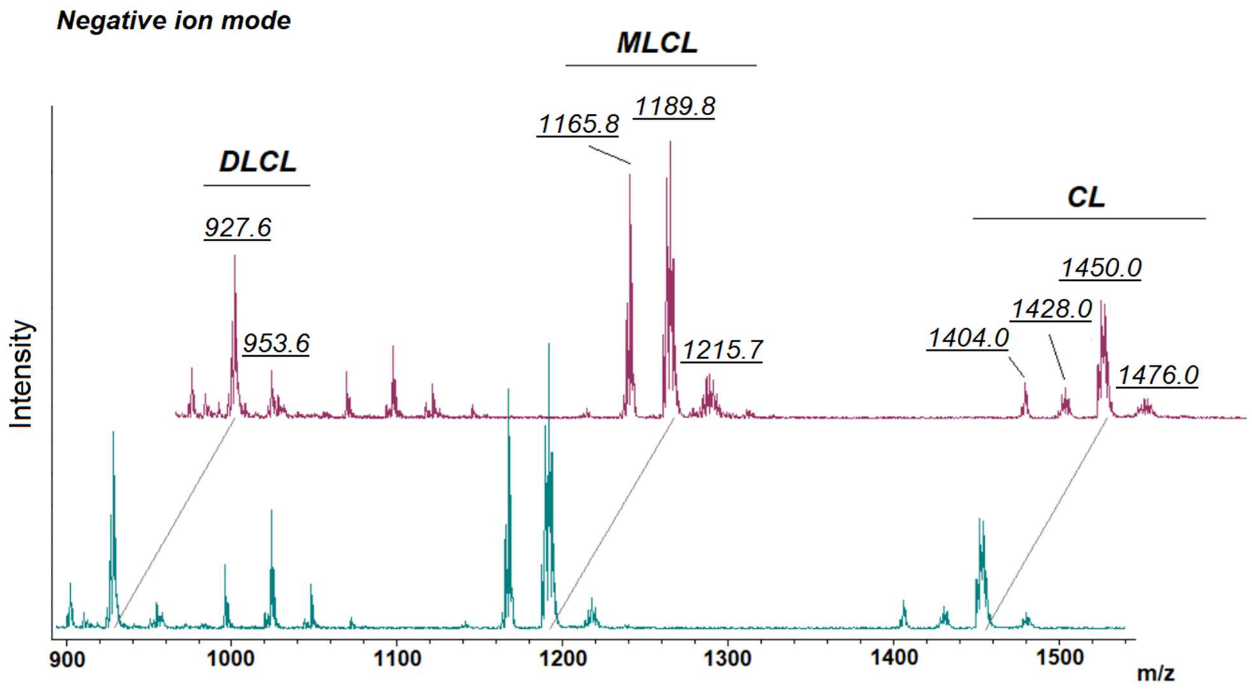


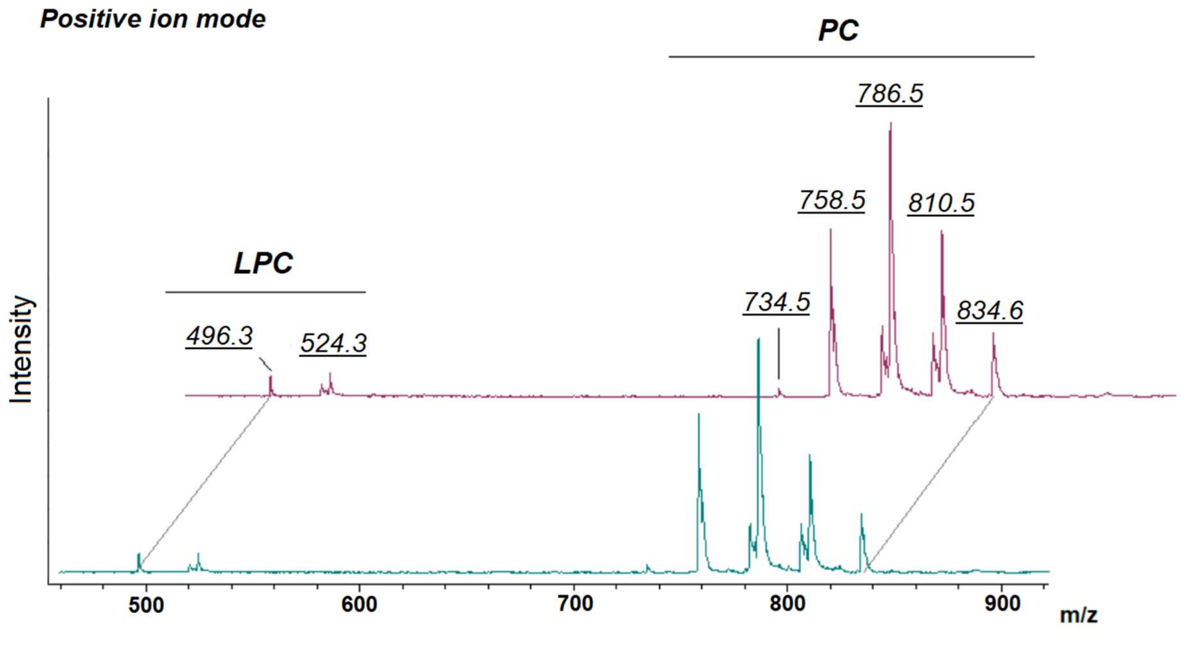


**FIGURE S2. Lipid profiles of TazKD + saline and TazKD + SS-31 mitochondria in negative and positive ion modes MALDI-TOF/MS.** Cardiac mitochondria were isolated from 6-month-old mice Taz$^{KD}$ + saline (violet) and Taz$^{KD}$ + SS-31 (green) and lipids were analysed as described in Methods. Lipid assignments for main signals are indicated.

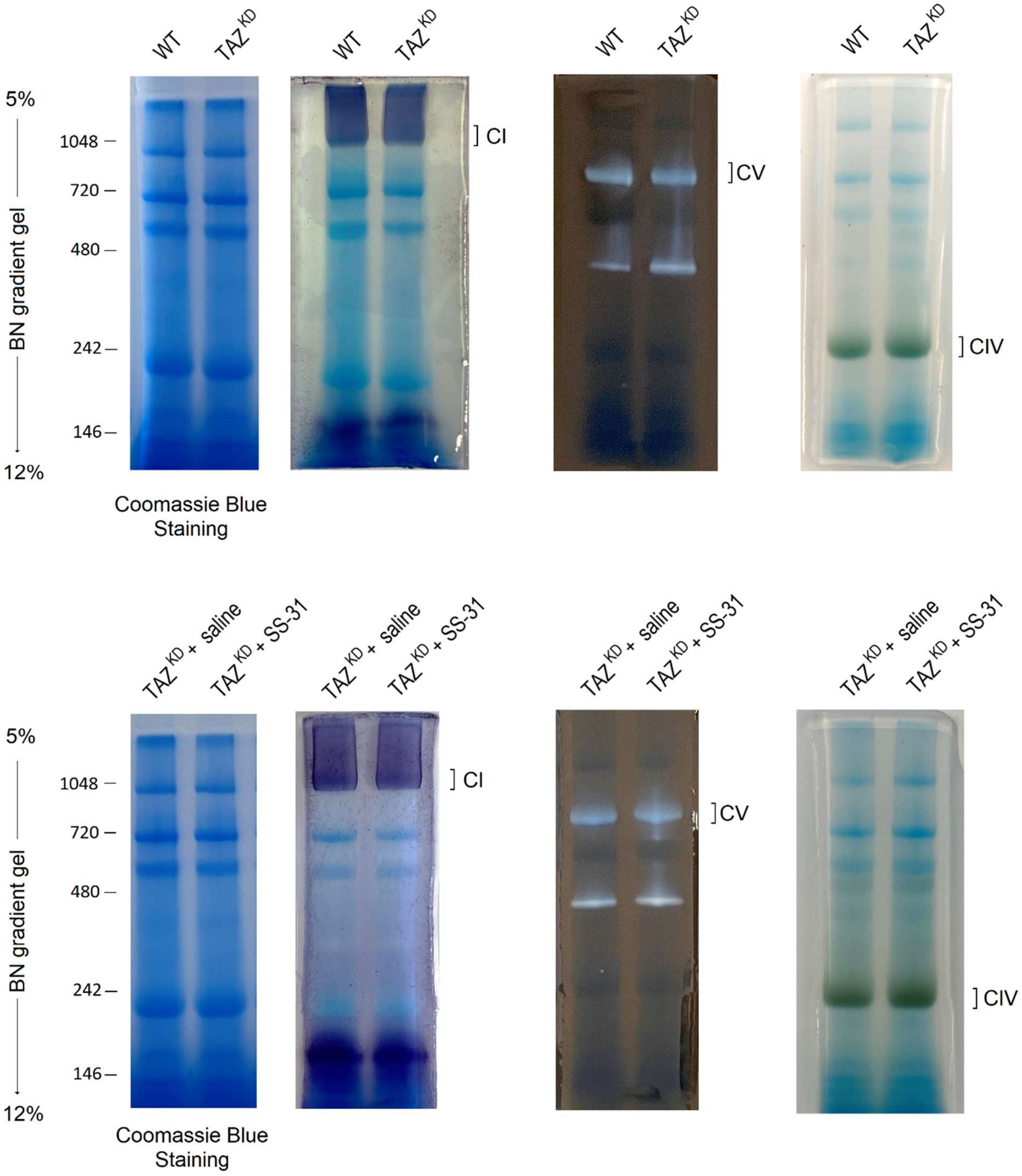


**<u>FIGURE S3. In-gel activity of OXPHOS complexes</u>**. Blue-native (BN) PAGE was performed as previously described (*Schagger H and von Jagow 1991 Anal. Biochem. 199:223-31*) with minor modifications. Mitochondrial proteins (50 µg) were solubilized in the presence of 0.4% dodecylmaltoside and subjected to electrophoresis. After runs, in-gel activity staining was performed for complex I, V, and IV, as previously described (*Jha P., Wang X. and Auwerx J. 2016 Curr Protoc Mouse Biol. 6(1): 1–14.).* Acquisition and quantification of band intensities of histochemical reactions were performed using the Chemidoc Touch Imaging System (Bio-Rad, California, USA). Densitometric analysis was performed using Image Lab (Bio-Rad, California, USA).

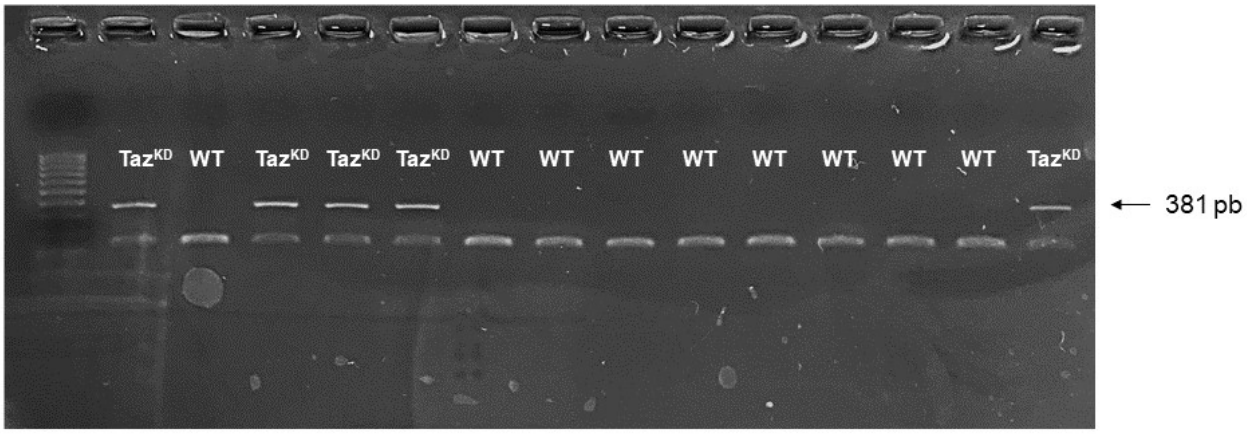


**FIGURE S4. Taz$^{KD}$ genotyping.** Representative PCR results from amplification of genomic tail DNA. Mice positive for the transgene were identified by the 381 pb DNA product.

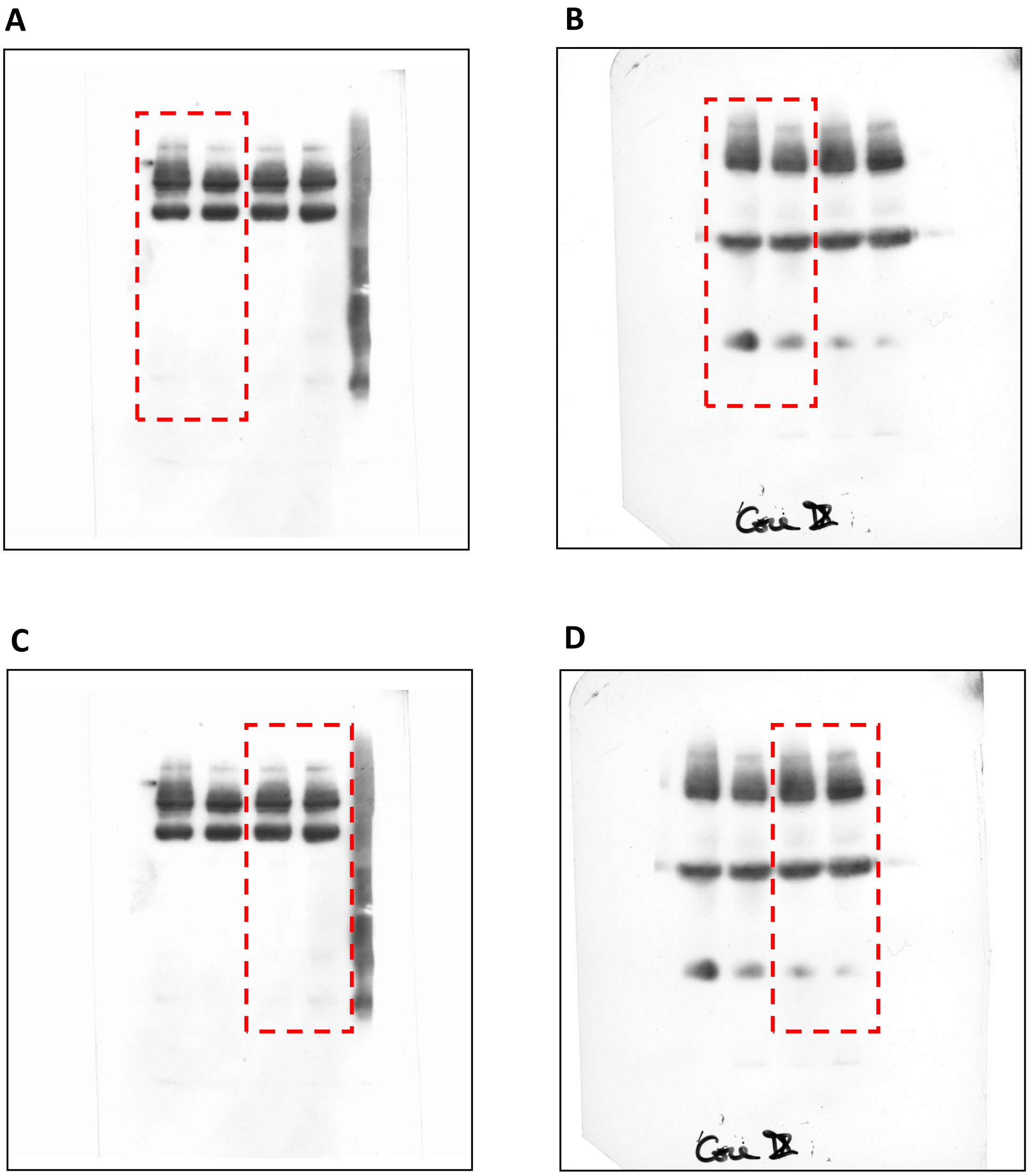


**FIGURE S5. Respiratory chain supercomplex analysis.** (A) Full scan of western blotting analysis shown in Figure 5A left panel. (B) Full scan of western blotting analysis shown Figure 5A right panel. (C) Full scan of western blotting analysis shown Figure 5D left panel. (D) Full scan of western blotting analysis shown Figure 5D right panel. Cropped areas are marked by red colour.

| | Complex I | p-value | Complex V | p-value | Complex IV | p-value |
|---|---|---|---|---|---|---|
| **WT** | 100.0 (± 21.4) | 0.43 | 100.0 (± 6.5) | 0.50 | 100.0 (± 12.1) | 0.31 |
| **Taz$^{KD}$** | 86.3 (± 19.5) | | 95.4 (± 8.9) | | 108.1 (± 0.3) | |
| **Taz$^{KD}$ + saline** | 100.0 (± 2.0) | 0.68 | 100.0 (± 30.8) | 0.39 | 100.0 (± 0.1) | 0.35 |
| **Taz$^{KD}$ + SS-31** | 107.3 (± 26.8) | | 83.0 (± 0.8) | | 87.1 (± 24.5) | |

**TABLE S1. In-gel activity analyses of OXPHOS complexes**. In-gel complexes activity levels estimated by band densitometry in Taz$^{KD}$ and in Taz$^{KD}$ + SS-31 are expressed as percentages of WT and Taz$^{KD}$ + saline, respectively. All values are means of arbitrary densitometric units ± SD expressed as percentages (n=3/group). P-values were determined by Student's t-test.
